# Exploring the Links between Physical Activity, Emotional Regulation, and Mental Well-Being in Jordanian University Students

**DOI:** 10.3390/jcm13061533

**Published:** 2024-03-07

**Authors:** Mohammad Al-Wardat, Chiara Salimei, Hassan Alrabbaie, Mohammad Etoom, Malak Khashroom, Chantelle Clarke, Khader A. Almhdawi, Talitha Best

**Affiliations:** 1 Department of Rehabilitation Sciences, Faculty of Applied Medical Sciences, Jordan University of Science and Technology, P.O. Box 3030, Irbid 22110, Jordan; msetoom@just.edu.jo (M.E.); khader@just.edu.jo (K.A.A.); 2 Department of Clinical Science and Translational Medicine, University of Rome Tor Vergata, 00133 Rome, Italy; chiara.salimei@gmail.com; 3 School of Rehabilitation Science, McMaster University, Hamilton, ON L8S 4L8, Canada; alrabbah@mcmaster.ca; 4 Department of Educational Psychology, Faculty of Educational Sciences, University of Jordan, Amman 11942, Jordan; mtkhashroom20@ams.just.edu.jo; 5 NeuroHealth Lab, Appleton Institute, School of Health, Medical and Applied Sciences, Central Queensland University, Brisbane 4000, Australia; chantelle.clarke@cqumail.com (C.C.); t.best@cqu.edu.au (T.B.)

**Keywords:** physical activity, emotional regulation, depression, anxiety, stress, students

## Abstract

**Background:** University students face multiple stressors that negatively impact their mental well-being. Effective emotional regulation and physical activity are crucial for mood management and overall health. This study explored the connection between physical activity, emotional regulation, and mental health symptoms (depression, anxiety, and stress) in Jordanian university students. **Methods**: A cross-sectional online survey involved 416 students (146 male and 270 female) from Jordanian universities. The survey covered demographics, physical activity (International Physical Activity Questionnaire), emotional regulation strategies (Emotion Regulation Questionnaire), and mental health symptoms (Depression Anxiety Stress Scales). Pearson’s correlations examined relationships, and ANOVA compared differences in ‘low’, ‘medium’, and ‘high’ physical activity groups. **Results**: Greater use of expressive suppression was correlated with increased anxiety symptom severity (*p* = 0.029). Although physical activity levels were not significantly related to emotional regulation, the ‘high’ physical activity group reported lower depression (*p* < 0.001) and anxiety symptom severity (*p* < 0.001) than the ‘low’ and ‘medium’ groups. **Conclusions**: Increased physical activity and emotional expression suppression are independently associated with improved mental well-being in Jordanian university students. This study underscores the importance of integrating physical activity and emotional expression strategies to support student well-being.

## 1. Introduction

Physical activity (PA) has a positive impact on mental health and emotional regulation (ER) [1]. Indeed, PA has a beneficial effect on cognitive abilities such as memory and attention [2] and emotional states, particularly by reducing symptoms and the risk of developing anxiety or depression [2,3]. The beneficial effect of regular exercise is often attributed to the production of endorphins, which contribute to mood regulation and enhance the function of the prefrontal cortex, a region of the brain responsible for ER and decision-making [4]. Moreover, PA provides a mechanism for stress relief and can assist individuals in regulating their emotions by allowing them to process and manage their feelings [5]. Additionally, PA can increase self-esteem and promote feelings of self-esteem, which positively influence both ER and mental health [1]. However, the association between PA, mental status, and ER can be complex and individualized. External and internal factors such as genetics, personal circumstances, and lifestyle can also play a role in determining one’s mental and emotional well-being [6]. Consequently, ER is the capacity to adaptively manage and control emotional responses in socially acceptable ways [7]. It involves recognizing, understanding, and employing strategies to navigate diverse situations, promoting mental resilience and well-being aligned with personal goals and values [7].

Indeed, PA and ER are closely related [8]. One strategy for ER is reappraisal, which involves changing the way of interpreting and responding to a situation [9]. Emotional reappraisal involves taking a step back and reframing a situation in a more positive or constructive way [9]. This can be especially helpful in reducing stress and negative emotions. Indeed, PA may used as a form of reappraisal [10]. For example, when experiencing anxiety or stress, engaging in PA, such as walking or any form of exercise, can aid in redirecting one’s attention, thereby facilitating a more favorable reframing of the situation [10]. This alteration in focus can further help in releasing accumulated energy and lowering stress levels. Incorporating PA into an ER strategy can aid mood enhancement and stress reduction [10]. PA can serve as a reappraisal technique, providing an individual with the ability to exert control over their emotions and promoting improved and more effective management of emotional states [5,10].

PA and expressive suppression are also related to ER [11]. Expressive suppression refers to the act of inhibiting or hiding one’s emotional expressions [12]. In fact, previous studies have shown that expressive suppression can lead to an increase in negative emotions and physiological arousal [12,13]. PA serves as an alternative to expressive suppression as a means of regulating emotions [11]. Despite the emotional inhibition, PA allows individuals to express and release their emotions in a healthy and constructive manner. This can lead to a decrease in negative emotions and an improvement in mood (depression, anxiety, and stress) [14]. This suggests that incorporating PA into an individual’s ER strategy may be advantageous for managing challenging emotional states and decreasing the reliance on expressive suppression as a coping mechanism [10]. However, expressive suppression can have both positive and negative effects, depending on the situation. In some cases, suppressing emotions may be necessary for social and professional reasons, but it is important to find a balance and engage in activities, such as PA, that promote emotional well-being.

Evidence explaining the relationship between brain structure and ER strategies has been provided by neuroimaging research. Structural brain plasticity measured by cortical thickness reflects the distance between the external surface of gray matter and the inner surface of white matter. These studies examined the association between cortical thickness and variations in the utilization of two ER strategies: cognitive reappraisal and expression suppression. Interestingly, both ER strategies exhibit marked correlations of cognitive reappraisal utilization with neural activity in specific regions of the prefrontal cortex, such as the ventromedial prefrontal, dorsolateral prefrontal, and dorsal anterior cingulate cortices [15,16,17,18]. The research indicates that cortical thinning observed in the left ventrolateral and dorsolateral prefrontal cortex among adolescent females is notably linked to increased [19]. Additionally, a significant link was found between expression suppression and other brain structures represented by the superior frontal gyrus, inclusive of the medial prefrontal cortex, precuneus, and parahippocampal gyrus [20]. These findings highlight robust empirical evidence supporting the association between cortical thickness and ER strategies. Despite this extensive evidence, there remains variability in the correlation between ER strategies and cortical regions across diverse brain regions among young participants, necessitating immediate investigation.

PA beneficially impacts both brain structures and functions [21,22,23]. A study found decreases in the thickness of the prefrontal cortex and parahippocampal cortex in physically active children compared to inactive children [24]. In addition, increased PA levels (moderate to high levels) among adults are associated with a thicker cortex in the left hemisphere’s temporal pole and superior frontal gyrus, regions that decline with age [25]. Despite these beneficial effects of PA, a previous study revealed no significant alterations in the brain activation within the anterior cingulate cortex (ACC) among participants during a 9-month follow-up [26]. Similarly, the cortical thickness in response to exercise was not significantly changed after a 5-year PA intervention [27]. This is not surprising; indeed, the participant-specific factors and inconsistencies in the PA protocols and measurement methodologies contributed to these variations. University students, in the stage of early adulthood, exhibit development trends in the cerebral gray matter cortex, transitioning from cortical thickening in childhood to cortical thinning [28]. Given that cortical thickness may effectively predict the utilization of ER strategies in early adulthood, thinner dimensions of both left dlPFC and vlPFC may utilize cognitive reappraisal, while thinner superior frontal gyrus thickness is positively associated with expression suppression in females, although negatively correlated in males [18,19]. The role of cortical thickness in mediating the association between PA and ER among different populations needs further investigation.

Conversely, physical inactivity has also been linked to ER [5,10]. Indeed, sedentary behavior can lead to negative changes in mood, such as feelings of anxiety, depression, and irritability [8]. Additionally, physical inactivity has been associated with decreased levels of brain neurotransmitters, such as serotonin and norepinephrine, which play important roles in regulating mood and emotions [29]. Moreover, physical inactivity can lead to physical health problems, such as obesity and cardiovascular disease, which can have a negative impact on mental health and further contribute to difficulties in ER [30]. Therefore, this study aimed to investigate the relationship between PA levels, emotional regulation, and mental health symptoms (depression, anxiety, and stress) among Jordanian University students. We hypothesize that there is a significant correlation between levels of PA among Jordanian University students and their emotional regulation skills. Furthermore, we predict that higher levels of PA will be associated with lower mental health symptoms, including reduced depression, anxiety, and stress, suggesting a positive impact of PA on the emotional well-being of university students in Jordan.

## 2. Materials and Methods

### 2.1. Design, Sampling, and Participants

This study was a cross-sectional online survey carried out in accordance with the STROBE (Strengthening the Reporting of Observational Studies in Epidemiology) guidelines [31]. The study participants were undergraduate students from both public and private universities in Jordan. The survey was conducted from September to December 2022 and was distributed via social media. The survey started with an introduction about the study aim and the study consent form. The initial survey question concerned whether the student was interested in participating or not. Those who answered ‘no’ were automatically excluded from further participation. The study was approved by the Institutional Review Board (IRB) at Aqaba University of Technology (AUT-22-012-03).

### 2.2. Inclusion and Exclusion Criteria

The inclusion criteria were being an undergraduate student studying in a public or private university, age between 18 and 30, and a full-time enrolment in one of the Jordanian public or private institutions. Exclusion criteria included postgraduate students, a part-time enrolment, use of psychiatric medication, a history of severe physical or mental disorders, and students who could not communicate or understand the Arabic language.

### 2.3. Sample Size Calculation

According to the statistics of the Jordanian Ministry of Higher Education and Scientific Research, there are 332,413 students. Based on the probability sampling method and based on the sample size calculation of a 95% confidence level with a margin error of ±5%, the required sample size was 384 participants [32].

### 2.4. Outcome Measures

The survey questionnaire used in this study consisted of a section on sociodemographic information, which included data on age, gender, height, weight, body mass index (BMI), smoking status, type of university, GPA, academic year level, and any chronic diseases. The survey also included the use of valid and reliable standardized Arabic versions of the following questionnaires:

Depression Anxiety Stress Scale (DASS): This measure was used to assess the level of mental health symptoms among study participants. The measure has three subscales covering depression, anxiety, and stress and is considered valid and reliable. The measure uses a frequency rating scale (never–all the time) to rate statements such as “I was aware of dryness of my mouth” and “I found it difficult to relax”. A higher DASS score suggests a higher level of mental health symptoms. The cut-off points indicating the presence of mild or greater mental health symptoms are 10 for depression, 8 for anxiety, and 15 for stress [33,34]. The DASS scale has been validated and proven reliable for use in the Arabic language [35]. In this study, the Cronbach’s alpha were (α = 0.56) for depression, (α = 0.46) for anxiety, and (α = 0.41) for stress.

The International Physical Activity Questionnaires Short Form (IPAQ-SF) was used to assess physical activity levels. The IPAQ-SF is a self-reported questionnaire that categorizes participants into high, moderate, or low physical activity levels based on seven questions that measure the duration and frequency of physical activity performed over the previous seven days. Additionally, the IPAQ-SF measures the time spent sitting and calculates and scores the duration of vigorous, moderate-intensity, and walking activities in MET min/week units [36]. The IPAQ-SF questions are allocated across four categories of physical activities: transportation, work, domestic, and leisure. The scoring is expressed in metabolic equivalent minutes (MET) per week, computed by multiplying the MET values (3.3 for low physical activity such as walking, 4 for moderate physical activity, and 8 for high physical activity) by the total days and minutes devoted to each specific activity [37]. IPAQ-SF has shown high reliability and moderate validity among young and middle-aged adults and has been translated and validated into Arabic [36]. The IPAQ-SF has also demonstrated good test-retest reliability and high internal consistency, as measured by Cronbach’s alpha, among adult individuals [38].

The Emotion Regulation Questionnaire (ERQ) is a self-administered tool that evaluates the frequency of emotion regulation strategies, specifically cognitive reappraisal and expressive suppression, used by individuals [39]. The ERQ consists of two subscales, reappraisal (6 items) and suppression (4 items), and participants respond to each item on a 5-point Likert scale (1 = strongly disagree to 5 = strongly agree). The ERQ has been proven to be valid and reliable and has been translated and used in the Arabic population [39]. In this study, Cronbach’s alpha was (α = 0.79) for the reappraisal scale and (α = 0.64) for the suppression scale.

Before distributing the final version of the survey, a panel of experts reviewed the draft version, and it was tested on 10 students to ensure its comprehensibility. The pilot participants reported that it took them an average of 20 min to complete the survey, and they provided positive feedback with no reported difficulties in understanding the questions.

### 2.5. Data Analysis

Data analyses were performed using SPSS version 26 (SPSS, Inc., Chicago, IL, USA). Data assumption and normality testing for parametric and non-parametric analysis were conducted using the Kolmogorov–Smimov test, with no extreme outliers greater than 3SD identified. Participant characteristics were examined using descriptive statistics. Potential background demographics of gender, age, university type, years at university, smoking status, and GPA were considered as potential covariates for any significant differences in PA. There were no significant covariates for the categorical and ordinal data included in the analysis. Pearson’s product–moment correlations were conducted to examine the relationships between BMI, emotional regulation, and mood variables. To assess the differences in self-reported severity of depression, anxiety, stress, and emotional regulation among three physical activity level groups (‘low’, ‘medium’, and ‘high’), One-Way Analysis of Variance (ANOVA) omnibus tests were conducted. Subsequently, post hoc Bonferroni tests were conducted to further examine the specific group differences. All statistical analyses maintained a significance level of *p* < 0.05.

## 3. Results

Participant characteristics are described in Table 1. As shown, approximately two-thirds of participants were female (65%) and over 20 years old (67%). Approximately three-quarters attended a public university (74.8%). Over half (57%) were in their second year, and the majority indicated that their Grade Point Average (GPA) was ‘Very Good’ (88.5%). The majority did not smoke (78.1%) or have a chronic health disease (99.8%) and were categorized as engaging in a ‘high’ level of physical activity per week (79.1%). The prevalence of moderate to severe levels of anxiety, depression, and stress was 76.2%, 54.1%, and 41.6%, respectively.

As shown by Table 2, significant positive relationships were found between expressive suppression and BMI, *p* = 0.012; cognitive reappraisal, *p* ≤ 0.001; and anxiety, *p* = 0.029, and between anxiety and depression, *p* < 0.001. This indicates that those who are more likely to suppress their emotions are also more likely to have a greater BMI, use cognitive reappraisal to regulate emotions, and experience greater symptoms of anxiety. Further, those with greater symptoms of anxiety are also more likely to experience greater symptoms of depression.

**Figure 1:**
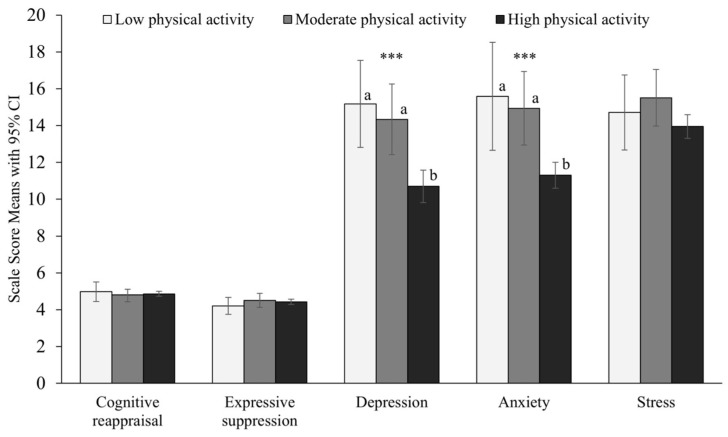
Means, 95% Confidence Intervals, and One-Way Analyses of Variance in emotional regulation and mood variables by physical activity group. *** *p* < 0.001. ^a,b^ indicates the groups that differed significantly from one another. Significant group differences were identified for depression *F*(2) = 8.90, *p* < 0.001, Cohen’s *d* = 0.41 and anxiety *F*(2) = 11.65, *p* < 0.001, Cohen’s *d* = 0/48.

As shown in Figure 1, One-Way Analysis of Variance omnibus tests showed that physical activity groups differed significantly in terms of self-reported severity of depression and anxiety with small effect sizes. Post hoc Bonferroni tests showed that the ‘high’ physical activity group reported significantly lower severity of symptoms for both depression and anxiety compared to both the ‘low’ and ‘medium’ physical activity groups. No significant differences were found between physical activity groups on stress or emotional regulation variables.

## 4. Discussion

The current study explored the relationships between PA levels, ER strategies, and mental health symptoms of depression, anxiety, and stress among Jordanian university students. Participants with high PA levels showed significantly lower symptoms of anxiety and depression compared to those with moderate or low PA. Furthermore, expressive suppression as an ER strategy was correlated with higher BMI and higher anxiety symptoms. There was no significant relationship between PA levels and the use of ER strategies. These findings highlight the complex relationships between ER and PA with mental health in a student population. These findings highlight the multifaceted connections that exist between ER, PA, and mental health within a student demographic. The implications of these results extend to understanding the intricate dynamics influencing mental well-being among university students, shedding light on potential opportunities for intervention and support in promoting holistic health and wellness within this population.

Overall, participants showed high levels of mental health symptoms, which is consistent with emerging data of student cohorts across disciplines and departments [40]. The prevalence rates of anxiety, depression, and stress in our study align with the average prevalence rate from a previous report that showed that college students have a moderate level of depression and stress and a severe level of anxiety [40,41,42]. In the current study, anxiety was significantly correlated with the expressive suppression ER strategy. This high level of mental health symptoms in this sample may explain the high frequency of using expressive suppression as an ER strategy. Conversely, it is plausible that greater use of emotional suppression is related to greater symptoms of anxiety due to active suppression and avoidance. Emotion expression suppression may not be evident in low-stress conditions [43], which may explain the greater use of this ER strategy in our sample, as university students are known to experience high levels of stress. The students with high PA levels exhibited significantly lower levels of anxiety and depression compared to those with low or moderate PA levels. Despite the absence of significant relationships between PA levels and ER strategies, there was a relationship between PA and expressive suppression with mental health symptoms. This is perhaps not surprising as anxiety in college students may be multifaceted due to physical, emotional, biological, relationship, and performance-related stressors. Here, we could recommend PA programs for stress and other mental health symptoms, and therefore, expressive suppression ER in young healthy participants. The small to moderate Cohen’s effect size in the ANOVA analysis implies that the interventions based on high levels of PA may result in true and real-world effectiveness [44].

PA level was not related to ER strategies, which is contradictory to our expectations. A previous study [45] found significant differences in the use of the cognitive appraisal but not suppression ER strategy among high, medium, and low PA groups. In other words, the cognitive reappraisal ER strategy was commonly used in the high PA group compared to the low PA group. In our report, although highly physically active participants experienced lower levels of anxiety and depression, they did not exhibit better ER strategies represented by more cognitive reappraisal and less emotion suppression. Our findings highlight the significance of ER strategies in the context of individual and contextual factors, as well as mental and emotional well-being [46]. Specifically, the observed correlation between the expressive suppression ER strategy, BMI, and anxiety underscores the potential role of personality traits in influencing ER. Future studies should delve deeper into individual state or trait-based characteristics of mood and emotional regulation to explore complex cognitive and psychological factors associated with ER strategies. Understanding the interrelationship between these variables not only enhances our theoretical framework but also has practical implications for interventions aimed at improving both emotional well-being.

Our findings revealed a significant positive correlation between cognitive reappraisal and expressive suppression ER strategies. This correlation suggests that participants may strategically utilize different ER strategies based on the demands of the context to achieve emotional balance. For instance, in the university environment, a student might employ cognitive reappraisal to manage internal anxiety while simultaneously using expressive suppression to navigate dissatisfying aspects of academic life, such as the challenges posed by online learning [47]. This observation aligns with the notion that individuals flexibly adapt their emotional regulation strategies based on the specific demands of the situation [48,49]. The interplay between cognitive reappraisal and expressive suppression highlights the dynamic nature of emotional regulation, emphasizing its adaptability to diverse contexts and stressors. Further research exploring the nuanced interaction between specific ER strategies in various situations can enhance our understanding of the complexity and fluidity of emotional regulation processes.

The COVID-19 pandemic has significantly impacted the lives of young university students, resulting in numerous stressors and demanding adaptive coping strategies [50]. As the pandemic disrupted traditional modes of learning and social interaction, students faced extraordinary challenges ranging from academic uncertainties to heightened concerns about personal health and mental health [51]. The sudden transition to remote learning, coupled with the pervasive sense of uncertainty regarding future prospects, exacerbated existing stressors and elicited novel sources of anxiety among students [52]. In addition, COVID-19 restrictions impaired the students’ activities and participation. Therefore, the social isolation forced by lockdown measures deprived students of vital support networks, aggravating feelings of loneliness and disconnection [53]. In response to these multifaceted stressors, students employed diverse coping mechanisms, ranging from seeking social support through virtual channels to engaging in mindfulness practices and self-care routines [54]. However, the efficacy of these coping strategies varied widely, with some students adapting to the rapidly evolving circumstances [54,55]. Through targeted interventions such as mental health counseling, peer support groups, accessible resources for stress management, and encouraging the student to practice regular PA, universities can empower students to navigate the challenges posed by crisis.

### Study Limitations

The cross-sectional design of this study limits the ability to establish causality between physical activity, ER, and mental health symptoms. The participant sample displayed a tendency towards reporting ‘high’ levels of physical activity, which suggests a potential self-selection bias where those interested in physical activity may have been more inclined to complete the survey. Social desirability bias might also have led to overreported PA levels. To counteract these biases, future research can incorporate objective measures like accelerometers for more accurate activity data and adopt random sampling techniques. More detailed awareness of contextual experiences of students in their mental health and data collection timeframes may also prove to be helpful, as sleep routines and study habits are also related to health behaviors, physical activity, and mental well-being, which were not considered in this study [56]. It is important to note that our study did not investigate other lifestyle variables such as drug use, alcohol consumption, and eating and sleeping habits. These factors have the potential to influence and constrain the breadth of our findings, possibly introducing confounding variables. We encourage future studies to explore a wider spectrum of lifestyle factors to gain a more comprehensive understanding of their potential impact on the observed outcomes. Investigating various dimensions of lifestyle, including drug use, alcohol consumption, and dietary habits, will enhance the comprehension of the intricate interplay between lifestyle choices and mental and emotional well-being.

## 5. Conclusions

A higher PA level is associated with lower mental health symptoms such as depression, anxiety, and stress compared to low or moderated levels of PA. There was no significant relation between PA and the use of ER strategies. However, it is crucial to note that there was no direct relationship between PA and expressive suppression ER strategy despite both being associated with anxiety. In this context, interventions promoting regular PA could prove beneficial in alleviating stress and, consequently, improving ER among young and healthy participants. In addition, intervention-based support (from therapists, mentors, and educators) that provides a warm and responsive approach and aids progress and mastery in handling anxiety and conflicting external pressures is likely to promote ER within the structure and context of a warm relationship.

## Figures and Tables

**Table 1:** Frequencies and percentages for participant characteristics.

Variables	Numbers	Percentage %
Sex		
Male	146	35%
Female	270	65%
Age		
>20 years old	279	67.1%
<20 years old	137	32.9%
University		
Public	311	74.8%
Private	105	25.2%
Academic Year		
1st year	89	21.4%
2nd year	237	57.0%
3rd year	68	16.3%
4th year	17	4.1%
5th year	5	1.2%
GPA		
Satisfactory	8	1.9%
Good	23	5.5%
Very good	368	88.5%
Excellent	17	4.1%
Smoker		
Yes	91	21.9%
No	325	78.1%
Chronic disease		
Yes	1	0.2%
No	415	99.8%
Physical Activity Level		
Low	34	8.2%
Moderate	53	12.7%
High	329	79.1%

**Table 2:** Pearson’s product–moment correlations between body mass index (BMI), physical activity (PA), emotional regulation, and mood variables.

	Mean	SD	PA	Cognitive Reappraisal	Expressive Suppression	Depression	Anxiety	Stress
BMI	26.70	4.48	−0.01	0.00	0.12 *	0.02	−0.00	0.08
PA	3289.66	2961.06		−0.04	−0.02	−0.05	−0.02	0.03
Cognitive Reappraisal	4.85	1.29			0.30 ***	0.04	−0.01	−0.07
Expressive Suppression	4.42	1.35				0.09	0.11 *	−0.06
Depression	11.53	8.00					0.34 ***	−0.00
Anxiety	12.12	6.89						0.11

Significant correlations are denoted with asterisks, with the significance level indicated (* for *p* < 0.05, *** for *p* < 0.001).

## Data Availability

The data that support the findings of this study are available on request from the corresponding author, M.A.-W.

## References

[1] Sharma A., Madaan V., Petty F.D. (2006). Exercise for Mental Health. Prim. Care Companion J. Clin. Psychiatry.

[2] Mandolesi L., Polverino A., Montuori S., Foti F., Ferraioli G., Sorrentino P., Sorrentino G. (2018). Effects of Physical Exercise on Cognitive Functioning and Wellbeing: Biological and Psychological Benefits. Front. Psychol..

[3] Alwardat M., Schirinzi T., Di Lazzaro G., Sancesario G.M., Franco D., Imbriani P., Sinibaldi Salimei P., Bernardini S., Mercuri N.B., Pisani A. (2019). Association between Physical Activity and Dementia’s Risk Factors in Patients with Parkinson’s Disease. J. Neural. Transm..

[4] Daniela M., Catalina L., Ilie O., Paula M., Daniel-Andrei I., Ioana B. (2022). Effects of Exercise Training on the Autonomic Nervous System with a Focus on Anti-Inflammatory and Antioxidants Effects. Antioxidants.

[5] Zhang Y., Fu R., Sun L., Gong Y., Tang D. (2019). How Does Exercise Improve Implicit Emotion Regulation Ability: Preliminary Evidence of Mind-Body Exercise Intervention Combined with Aerobic Jogging and Mindfulness-Based Yoga. Front. Psychol..

[6] Tsuang M.T., Bar J.L., Stone W.S., Faraone S. (2004). V Gene-Environment Interactions in Mental Disorders. World Psychiatry.

[7] Gross J.J. (2015). Emotion Regulation: Current Status and Future Prospects. Psychol. Inq..

[8] Neta M., Harp N.R., Henley D.J., Beckford S.E., Koehler K. (2019). One Step at a Time: Physical Activity Is Linked to Positive Interpretations of Ambiguity. PLoS ONE.

[9] Troy A.S., Shallcross A.J., Brunner A., Friedman R., Jones M.C. (2018). Cognitive Reappraisal and Acceptance: Effects on Emotion, Physiology, and Perceived Cognitive Costs. Emotion.

[10] Perchtold S.C.M., Fink A., Rominger C., Weiss E.M., Papousek I. (2020). More Habitual Physical Activity Is Linked to the Use of Specific, More Adaptive Cognitive Reappraisal Strategies in Dealing with Stressful Events. Stress Health.

[11] Wang K., Yang Y., Zhang T., Ouyang Y., Liu B., Luo J. (2020). The Relationship Between Physical Activity and Emotional Intelligence in College Students: The Mediating Role of Self-Efficacy. Front. Psychol..

[12] Cutuli D. (2014). Cognitive Reappraisal and Expressive Suppression Strategies Role in the Emotion Regulation: An Overview on Their Modulatory Effects and Neural Correlates. Front. Syst. Neurosci..

[13] Yan C., Ding Q., Wang Y., Wu M., Gao T., Liu X. (2022). The Effect of Cognitive Reappraisal and Expression Suppression on Sadness and the Recognition of Sad Scenes: An Event-Related Potential Study. Front. Psychol..

[14] Edwards M.K., Rhodes R.E., Mann J.R., Loprinzi P.D. (2018). Effects of Acute Aerobic Exercise or Meditation on Emotional Regulation. Physiol. Behav..

[15] Beauregard M., Lévesque J., Bourgouin P. (2001). Neural Correlates of Conscious Self-Regulation of Emotion. J. Neurosci..

[16] Ochsner K.N., Bunge S.A., Gross J.J., Gabrieli J.D.E. (2002). Rethinking Feelings: An FMRI Study of the Cognitive Regulation of Emotion. J. Cogn. Neurosci..

[17] Urry H.L., Van Reekum C.M., Johnstone T., Kalin N.H., Thurow M.E., Schaefer H.S., Jackson C.A., Frye C.J., Greischar L.L., Alexander A.L. (2006). Amygdala and Ventromedial Prefrontal Cortex Are Inversely Coupled during Regulation of Negative Affect and Predict the Diurnal Pattern of Cortisol Secretion among Older Adults. J. Neurosci..

[18] Hermann A., Bieber A., Keck T., Vaitl D., Stark R. (2013). Brain Structural Basis of Cognitive Reappraisal and Expressive Suppression. Soc. Cogn. Affect. Neurosci..

[19] Vijayakumar N., Whittle S., Yücel M., Dennison M., Simmons J., Allen N.B. (2014). Thinning of the Lateral Prefrontal Cortex during Adolescence Predicts Emotion Regulation in Females. Soc. Cogn. Affect. Neurosci..

[20] Wang K., Huang H., Chen L., Hou X., Zhang Y., Yang J., Hao X., Qiu J. (2017). MRI Correlates of Interaction between Gender and Expressive Suppression among the Chinese Population. Neuroscience.

[21] Seal E., Vu J., Winfield A., Fenesi B. (2023). Impact of COVID-19 on Physical Activity in Families Managing ADHD and the Cyclical Effect on Worsening Mental Health. Brain Sci..

[22] Rovio S., Spulber G., Nieminen L.J., Niskanen E., Winblad B., Tuomilehto J., Nissinen A., Soininen H., Kivipelto M. (2010). The Effect of Midlife Physical Activity on Structural Brain Changes in the Elderly. Neurobiol. Aging.

[23] Boyke J., Driemeyer J., Gaser C., Büchel C., May A. (2008). Training-Induced Brain Structure Changes in the Elderly. J. Neurosci..

[24] Chaddock-Heyman L., Weng T.B., Loui P., Kienzler C., Weisshappel R., Drollette E.S., Raine L.B., Westfall D., Kao S.C., Pindus D.M. (2021). Brain Network Modularity Predicts Changes in Cortical Thickness in Children Involved in a Physical Activity Intervention. Psychophysiology.

[25] Gu Y., Beato J.M., Amarante E., Chesebro A.G., Manly J.J., Schupf N., Mayeux R.P., Brickman A.M. (2020). Assessment of Leisure Time Physical Activity and Brain Health in a Multiethnic Cohort of Older Adults. JAMA Netw. Open.

[26] Chaddock-Heyman L., Erickson K.I., Voss M.W., Knecht A.M., Pontifex M.B., Castelli D.M., Hillman C.H., Kramer A.F. (2013). The Effects of Physical Activity on Functional MRI Activation Associated with Cognitive Control in Children: A Randomized Controlled Intervention. Front. Hum. Neurosci..

[27] Pani J., Marzi C., Stensvold D., Wisløff U., Håberg A.K., Diciotti S. (2022). Longitudinal Study of the Effect of a 5-Year Exercise Intervention on Structural Brain Complexity in Older Adults. A Generation 100 Substudy. Neuroimage.

[28] Burzynska A.Z., Nagel I.E., Preuschhof C., Gluth S., Bäckman L., Li S.C., Lindenberger U., Heekeren H.R. (2012). Cortical Thickness Is Linked to Executive Functioning in Adulthood and Aging. Hum. Brain Mapp..

[29] Lin T.-W., Kuo Y.-M. (2013). Exercise Benefits Brain Function: The Monoamine Connection. Brain Sci..

[30] Park J.H., Moon J.H., Kim H.J., Kong M.H., Oh Y.H. (2020). Sedentary Lifestyle: Overview of Updated Evidence of Potential Health Risks. Korean J. Fam. Med..

[31] von Elm E., Altman D.G., Egger M., Pocock S.J., Gøtzsche P.C., Vandenbroucke J.P. (2008). The Strengthening the Reporting of Observational Studies in Epidemiology (STROBE) Statement: Guidelines for Reporting Observational Studies. J. Clin. Epidemiol..

[32] Krejcie R.V., Morgan D.W. (1970). Determining Sample Size for Research Activities. Educ. Psychol. Meas..

[33] Patrick J., Dyck M., Bramston P. (2010). Depression Anxiety Stress Scale: Is It Valid for Children and Adolescents?. J. Clin. Psychol..

[34] Lovibond P.F., Lovibond S.H. (1995). The Structure of Negative Emotional States: Comparison of the Depression Anxiety Stress Scales (DASS) with the Beck Depression and Anxiety Inventories. Behav. Res. Ther..

[35] Moussa M.T., Lovibond P., Laube R., Megahead H.A. (2017). Psychometric Properties of an Arabic Version of the Depression Anxiety Stress Scales (DASS). Res. Soc. Work Pract..

[36] Helou K., El Helou N., Mahfouz M., Mahfouz Y., Salameh P., Harmouche-Karaki M. (2018). Validity and Reliability of an Adapted Arabic Version of the Long International Physical Activity Questionnaire. BMC Public Health.

[37] Jenkinson C., Layte R. (1997). Development and Testing of the UK SF-12. J. Health Serv. Res. Policy.

[38] Citko A., Górski S., Marcinowicz L., Górska A. (2018). Sedentary Lifestyle and Nonspecific Low Back Pain in Medical Personnel in North-East Poland. Biomed. Res. Int..

[39] Kahwagi R., Zeidan R.K., Haddad C., Hallit R., Sacre H., Kheir N., Salameh P., Obeid S., Hallit S. (2021). Emotion Regulation among Lebanese Adults: Validation of the Emotion Regulation Questionnaire and Association with Attachment Styles. Perspect. Psychiatr. Care.

[40] Sivertsen B., Knudsen A.K.S., Kirkøen B., Skogen J.C., Lagerstrøm B.O., Lønning K.-J., Kessler R.C., Reneflot A. (2023). Prevalence of Mental Disorders among Norwegian College and University Students: A Population-Based Cross-Sectional Analysis. Lancet Reg. Health—Eur..

[41] Dalky H.F., Gharaibeh A. (2019). Depression, Anxiety, and Stress among College Students in Jordan and Their Need for Mental Health Services. Nurs. Forum.

[42] Al-Wardat M., Etoom M., Almhdawi K.A., Hawamdeh Z., Khader Y. (2024). Prevalence of Attention-Deficit Hyperactivity Disorder in Children, Adolescents and Adults in the Middle East and North Africa Region: A Systematic Review and Meta-Analysis. BMJ Open.

[43] Wang M., Saudino K.J. (2011). Emotion Regulation and Stress. J. Adult Dev..

[44] Pascoe M.C., Bailey A.P., Craike M., Carter T., Patten R.K., Stepto N.K., Parker A.G. (2021). Single Session and Short-Term Exercise for Mental Health Promotion in Tertiary Students: A Scoping Review. Sports Med. Open.

[45] Wu J., Zhu L., Dong X., Sun Z., Cai K., Shi Y., Chen A. (2022). Relationship between Physical Activity and Emotional Regulation Strategies in Early Adulthood: Mediating Effects of Cortical Thickness. Brain Sci..

[46] Teixeira J.R.B., de Sousa A.R., Silva Palma E.M., Moreira W.C., da Silva Santana T., Barreto N.M.P.V., de Moura M.A., Vergara-Escobar O.J., Fabián José O.Y., Souza Pereira G. (2022). Factors Associated with Emotion Regulation in Men with Internet Access Living in Brazil during the COVID-19 Pandemic. Int. J. Environ. Res. Public Health.

[47] Etoom M., Aldaher K.N., Abdelhaq A.A., Alawneh A., Alghwiri A.A. (2023). Distance Learning in Physiotherapy Education during the COVID-19 Pandemic: Students’ Satisfaction, Perceived Quality, and Potential Predictors of Satisfaction. Physiother. Theory Pract..

[48] Coakley K.E., Lardier D.T., Holladay K.R., Amorim F.T., Zuhl M.N. (2021). Physical Activity Behavior and Mental Health Among University Students During COVID-19 Lockdown. Front. Sports Act Living.

[49] Rodríguez-Romo G., Acebes-Sánchez J., García-Merino S., Garrido-Muñoz M., Blanco-García C., Diez-Vega I. (2022). Physical Activity and Mental Health in Undergraduate Students. Int. J. Environ. Res. Public Health.

[50] Moldovan F., Gligor A., Moldovan L., Bataga T. (2022). The Impact of the COVID-19 Pandemic on the Orthopedic Residents: A Pan-Romanian Survey. Int. J. Environ. Res. Public Health.

[51] Pandya A., Lodha P. (2022). Mental Health Consequences of COVID-19 Pandemic among College Students and Coping Approaches Adapted by Higher Education Institutions: A Scoping Review. SSM—Ment. Health.

[52] David I., Schatz E., Myroniuk T.W., Teti M. (2022). “COVID Is Another Layer of Problematic Things”: Change, Vulnerability, and COVID-19 among University Students. Int. J. Environ. Res. Public Health.

[53] Hwang T.J., Rabheru K., Peisah C., Reichman W., Ikeda M. (2020). Loneliness and Social Isolation during the COVID-19 Pandemic. Int. Psychogeriatr..

[54] Alkhawaldeh A., Al Omari O., Al Aldawi S., Al Hashmi I., Ann Ballad C., Ibrahim A., Al Sabei S., Alsaraireh A., Al Qadire M., Albashtawy M. (2023). Stress Factors, Stress Levels, and Coping Mechanisms among University Students. Sci. World J..

[55] Freire C., del Mar Ferradás M., Regueiro B., Rodríguez S., Valle A., Núñez J.C. (2020). Coping Strategies and Self-Efficacy in University Students: A Person-Centered Approach. Front. Psychol..

[56] Cahuas A., He Z., Zhang Z., Chen W. (2020). Relationship of Physical Activity and Sleep with Depression in College Students. J. Am. Coll. Health.

